# Mapping of Single-Base Differences between Two DNA Strands in a Single Molecule Using Holliday Junction Nanomechanics

**DOI:** 10.1371/journal.pone.0055154

**Published:** 2013-02-05

**Authors:** Camille Brème, François Heslot

**Affiliations:** 1 Laboratoire Pierre Aigrain, Unité Mixte de Recherche 8551 de l’Ecole Normale Supérieure, Paris, France; 2 Laboratoire Pierre Aigrain, Université Pierre et Marie Curie - Paris 6, Paris, France; 3 Laboratoire Pierre Aigrain, Université Paris Diderot - Paris 7, Paris, France; Université d’Evry val d’Essonne, France

## Abstract

**Objective:**

The aim of this work is to demonstrate a novel single-molecule DNA sequence comparison assay that is purely based on DNA mechanics.

**Methods:**

A molecular construct that contained the two homologous but non-identical DNA sequences that were to be compared was prepared such that a four-way (Holliday) junction could be formed by the formation of heteroduplexes through the inter-recombination of the strands. Magnetic tweezers were used to manipulate the force and the winding applied to this construct for inducing both the formation and the migration of a Holliday junction. The end-to-end distance of the construct was measured as a function of the winding and was used to monitor the behavior of the Holliday junction in different regions of the intra-molecular recombination.

**Main Results:**

In the appropriate buffer, the magnet rotation induces the migration of the Holliday junction in the regions where there is no sequence difference between the recombining sequences. In contrast, even a single-base difference between the recombining sequences leads to a long-lasting blockage of the migration in the same buffer; this effect was obtained when the junction was positioned near this locus (the site of the single-base difference) and forced toward the formation of heteroduplexes that comprise the locus. The migration blockages were detected through the identification of the formation of plectonemes. The detection of the presence of sequence differences and their respective mappings were obtained from the series of blockages that were detected.

**Significance:**

This work presents a novel single-molecule sequence comparison assay that is based on the use of a Holliday junction as an ultra-sensitive nanomechanism; the mismatches act as blocking grains of sand in the Holliday “DNA gearbox”. This approach will potentially have future applications in biotechnology.

## Introduction

Single molecule DNA sequencing is currently of great interest [1], [2], [3], [4]. The simpler task of single-molecule sequence comparison without sequencing, which is less powerful in scope, has attracted less attention even though it may be useful and more accessible.

**Figure 1:**
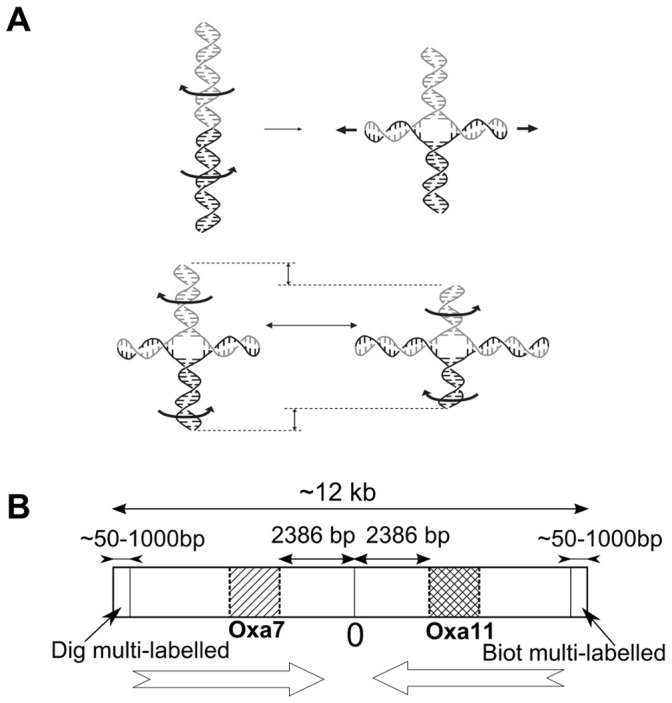
Sketch of a Holliday junction and its molecular construction. Fig. 1A: Conversion of a palindromic DNA molecule into a Holliday junction, in which the initial parental segments recombine to form heteroduplexes. If the recombining sequences differ by one base, two mismatches (one on each of the recombined arms) are formed. Fig. 1B: The molecule is nearly symmetric with respect to the center of the construct. In the opposite orientations, a first section of identical DNA segments is found near the center of each arm. This section is followed by an approximately 800-bp segment (oxa7 or oxa11) that exhibits a 5% difference between the sequences of the arms. The differing arm segments are followed by an identical DNA segment. The sequences of the regions that differ between the two arms are shown in the Sequence Information S1.

The comparison of DNA sequences is the hallmark of the identification of variants in a population. In this context, single molecule assays can potentially eliminate amplification biases and errors. In this study, we narrowed the focus to sequence comparison in the absence of sequencing. Although external probe hybridization-based single molecule assays may achieve single-base sensitivity, these require knowledge of the locus of interest (*e.g.*, [5]). The probeless hybridization-based mechanical unzipping of DNA [4], [6], [7] exhibits only limited sensitivity, in addition to mechanical instabilities and thermal fluctuations; moreover, the weakness of the base-pair interactions severely limit the accuracy of this method. The Holliday junction [8], [9], [10], [11], which is a four-armed structure in which two double-stranded DNA strands recombine to form hybrid double-stranded arms, is known to enable *in-vivo* DNA recombination. When used *in-vitro*, a Holliday junction (HJ) enables intra-molecular recombination between the two parental double-stranded DNA strands (Fig. 1A); however, a single base difference is able to impede the spontaneous random migration of the HJ [12], [13]. In this study, we address the feasibility of using a novel single-molecule DNA sequence comparison assay that is purely based on the mechanical properties of DNA and utilizes a Holliday junction as a mismatch-sensitive nanomechanical structure. The technique is probeless and relies on intra-molecular hybridization.

## Results

**Figure 2:**
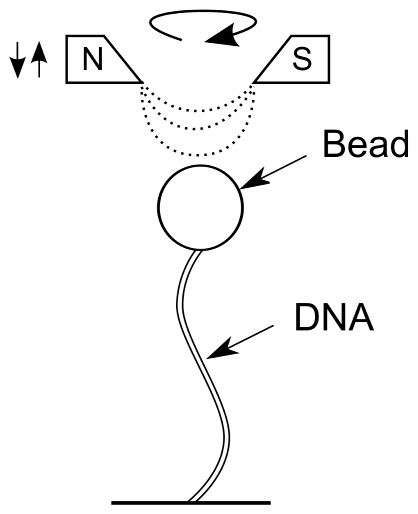
Sketch of the experimental configuration. The molecule is tethered between a glass surface and a paramagnetic bead. Multiple attachments at the extremities of the molecule ensure that the molecule is rotationally constrained. A pair of magnets, which are controlled above the sample by a motor, imposes the force and the rotation that is applied to the bead. Using video microscopy and concurrent image analysis of each video frame, the vertical and lateral positions of the tethered bead with respect to the surface of the sample are determined in real time. The extension of the molecule is deduced from the measurement of the average vertical position of the bead.

**Figure 3:**
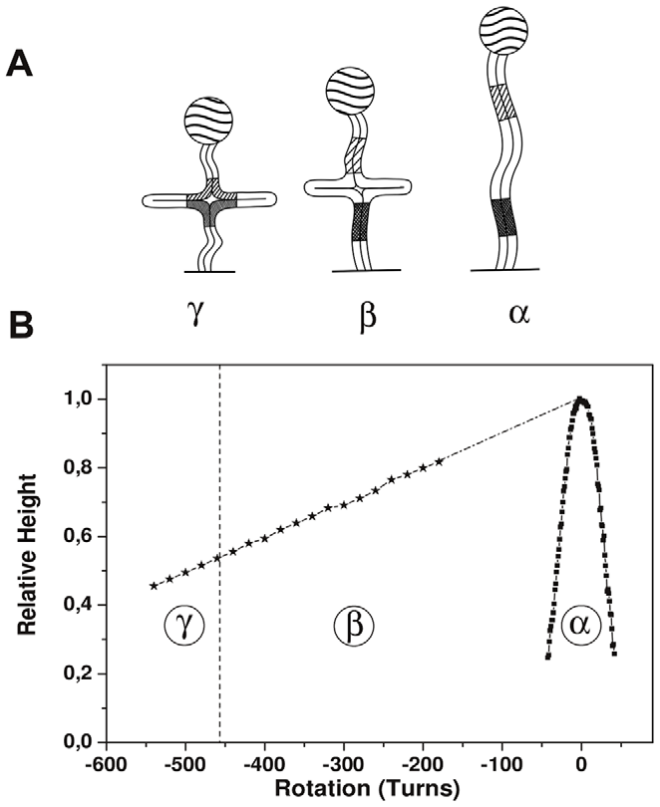
Molecular configuration and Holliday junction migration or blockage. Fig. 3A: Different configurations (α, β, and γ) of the molecular construct: (α) no HJ, (β) HJ but no mismatch, and (γ) HJ with mismatches in the lateral arms. A Holliday junction that is formed by micromanipulation can be moved by manipulating the winding applied to the construct. This movement is associated with a progression of the recombination. Fig. 3B: Data on the relative height (with respect to the height at R = 0) as a function of the rotation R in buffer Buff-A. The vertical traction force is approximately 0.35 pN. The bell-shaped curve near R = 0 corresponds to the formation of plectonemes with no HJ present (Fig. 3A-α). The data points in regions β and γ correspond to HJ migration (Fig. 3A-β and 3A-γ).

We used a magnetic tweezers assay [14] (Fig. 2) that involves the nanomechanics of a HJ [15], [16], [17]. The magnets are rotated to apply winding to the construction. The double-stranded DNA molecular construct is approximately 13 kb in length (see Fig. 1B and methods) and involves two localized test regions with a 5% relative sequence divergence (the naturally found beta-lactamase sequences oxa7 and oxa11 [18], [19], [20], which are associated with resistance to beta-lactams). The segments to be compared are combined in an opposite orientation within a single linear molecule. The construction is nearly symmetric with respect to its center, which allows the formation of a HJ (Figs. 1B and 3A). The traction force applied to the construction may be chosen from the typical range of 0.1 to 0.4 pN; in this range, the molecule is appreciably elongated and the symmetric formation of plectonemes is obtained [21], *i.e.*, the formation of plectonemes upon negative winding is similar to the formation of plectonemes upon positive winding, an indication that no partial unpairing of the double-stranded DNA is involved.

**Figure 4:**
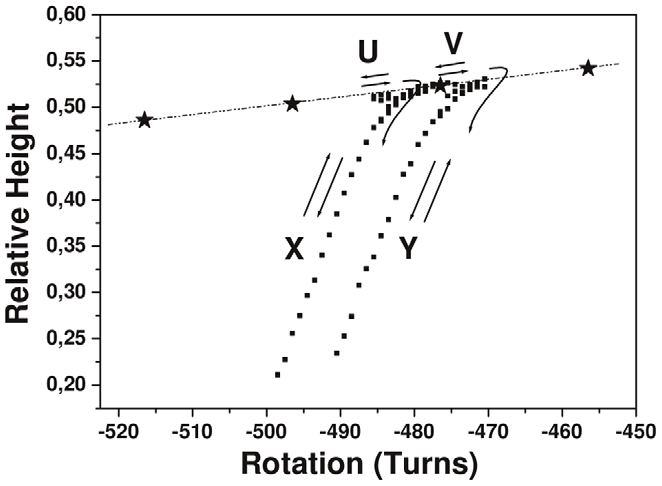
Holliday junction migration or blockage. Experimental data in Buff-B (squares). Some of the data shown in Fig. 3 (obtained in Buff-A and the selected range of rotation) are superimposed (stars). The curve-segments U, X, V and Y were obtained sequentially in Buff-B starting from R = −486; these curves were obtained using the same molecule that was used to obtain the data shown in Fig. 3. Curve-segment U was obtained for R in the range of −486 to −480, curve-segment X was initiated by decreasing R from −479, curve-segment V was obtained for R in the range of −479 −472, and curve-segment Y was initiated by decreasing R from −471. Curve-segments U and V correspond to HJ migration, whereas curve-segments X and Y correspond to a blocked HJ (toward more negative values of R) with the formation of plectonemes and are designated later in the text as “plectoneme-curve-segments”.

Two buffers were used in the experiments (see Methods): Buff-A (without magnesium ions) and Buff-B (with magnesium ions). Although the experiment was always started in Buff-A, this buffer was changed to Buff-B at a later stage of the experiment (discussed later). There is no HJ present at the initial stage of the experiment (in Buff-A). Fig. 3B shows an example of the measurement of the relative end-to-end distance Hrel of the construct (normalized to 1 at the zero rotation) as a function of the magnet rotation R (number of turns, either positive or negative). When limited winding is applied, the molecule displays the characteristic behavior of plectoneme formation [14], [15], [22], [23] because the corresponding bell-shaped “plectoneme formation curve” Cplecto is observed near R = 0 (region α in Fig. 3B). In fact, a bell-shaped plectoneme formation curve can also be obtained in Buff-B, *e.g.*, at the end of the experiment in Buff-B (see later). When a large negative unwinding (*e.g.*, −200 turns) is applied in Buff-A, a HJ can be observed with the characteristic feature of a constant slope of Hrel as a function of R (regions β and γ in Fig. 3B), which corresponds to reversible migration of the HJ [15]. This constant-slope behavior is similar in regions β and γ (although mismatches are expected to occur in region γ), which illustrates that the HJ migration in Buff-A appears unaffected by the mismatch formation. When the HJ is positioned in region γ and has mismatches in the side arms, the buffer is changed to Buff-B; all subsequent measurements in the experiment are performed in Buff-B. We focus below on a limited-range measurement sequence in Buff-B, which starts at approximately R = −486. The data are presented in Fig. 4. First, the curve-segment U is obtained for R in the range of −486 to −480. The curve-segment X was then initiated by first increasing R to −479 and then decreasing R from −479 to −500. The curve-segment V was obtained for R in the range of −479 to −472, and the curve-segment Y was then obtained by first increasing R to −471 and then decreasing R from −471 to −490. The curve-segments U and V approximately follow the constant slope curve that is associated with HJ migration. In addition, the curve-segments U and V were reversible, *i.e.* near-matching curve-segments were obtained if the rotation R was decreased or increased in the selected intervals. However, once the curve-segment X had been accessed, the curve-segment U was inaccessible, and once the curve-segment Y had been accessed, the curve-segments U, X, and V were inaccessible. The abrupt dependences of Hrel on R for the curve-segments X and Y (i) map approximately to the winding value at which a mismatch was expected to occur (see later) and (ii) may be superimposed through rescaling to obtain the characteristic shape Cplecto (see later), which hints that the curve-segment is the result of plectoneme formation due to the imposing of further negative turns in the presence of a migration blockage. The rescaling (see Methods) is linked to (i) the shorter tethering length because a part of the DNA is now in the lateral free arms of the junction and (ii) a rotation offset. Later in the text, we use the term “plectoneme-curve-segment” to designate the curve-segments with abrupt dependences of Hrel on R, such as the X and Y curve-segments. The events U, X, V, and Y illustrate that a mismatch in Buff-B behaves as a ratchet for HJ migration, *i.e.*, positive increments in R allow HJ motion, including the resumption of an existing mismatch, but the application of negative increments of R result in HJ blockage when a potential mismatch formation is encountered.

**Figure 5:**
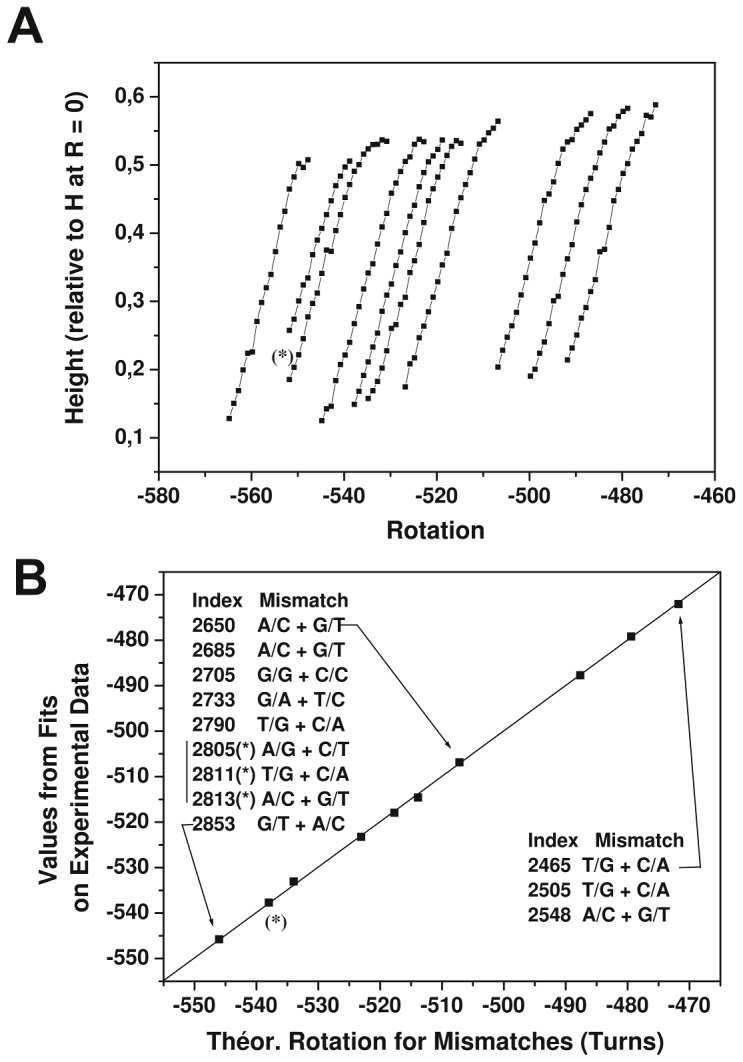
Experimental blockages and data treatment. Fig. 5A: Experimental data of the relative height (with respect to the height at R = 0) as a function of the rotation R for the plectoneme-curve-segments obtained in Buff-B in the region R = −465 to R = −545 using the process described in the methods section. The vertical traction force is approximately 0.18 pN. The plectoneme-curve-segments were obtained sequentially, starting from the left-most plectoneme-curve-segment on the figure (see Methods). Fig. 5B: The rotations Ri for the different blockages were determined using a non-linear fitting procedure (see Methods). The figure shows a plot of the experimental data-derived Ri (Turns) for the blockages depicted in Fig. 3A as a function of the expected blockage rotation (Turns) deduced from the sequence. A linear fit y = A+x for the data was obtained using the Origin software (OriginLab), which yielded A = 0.05, R-value = 0.9998, and Standard Deviation (SD) = 0.45. To independently obtain error estimates on the blockage values, a bootstrap method was used (see Methods); the SD obtained by bootstrap method was in the range of 0.5 to 0.6 turns. Inset of Fig. 5B: Indices of the expected mismatches in the construct and the mismatches formed. The asterisk (*) indicates the mismatches (over 8 bases) that were not resolved from their nearest (*)-labeled neighbors; only one blocking event was recorded with an uncertainty in the attribution. The “middle” (*) mismatch was arbitrarily assumed to be the one detected.

The following data were obtained from another molecule with the same construct to map multiple differences in the construct using a measurement sequence that does not assume any a priori knowledge of the existing sequence differences. A manipulation of the rotation was obtained through a series of progressive and partial go-and-return changes in the rotation starting from a given initial negative rotation, as described below. As described previously, a HJ is first formed in Buff-A. R was then adjusted to approximately −545, the buffer was changed to Buff-B, and all further measurements of the plectoneme-curve-segments for values of R from −545 to −465 were performed in Buff-B, as described in Methods. The data for the blockage events in the region corresponding to values of R from −465 to −545 are presented in Fig. 5A. The data comprise a succession of similar curve-segments with different horizontal rotation offsets. In addition, a trend of increasing maximum height as the rotation neared zero was observed. The rotation offsets Ri that correspond to the different windings at which the blockages occurred were determined using a non-linear fitting procedure (see Methods). Fig. 5B shows the values of Ri as a function of their expected position in the recombining sequences; the helical repeat of DNA (HRDNA) was assumed to be 10.45 base pairs/turn [24]. A linear fit y = A+x was performed, and the standard deviation of the experimental points with respect to the fit was approximately 0.5 turns, which corresponds to (0.5 * HRDNA/2) = 2.6 bases in the relative determination of the position of the mismatches. A correspondence between the predicted mismatch positions and the relative values of the blockages was thus obtained. A single-base difference generates a pair of mismatches. The combinations (A/G+C/T), (A/C+G/T), and (G/G+C/C) are present in the range of sequence explored in Fig. 5 and were detected; although the combination (A/A+T/T) was not present in this range, it was present and detected in another range (data not shown).

### Reproducibility of the Measurements and Typical Experimental Difficulties

A number of experimental difficulties were encountered (see Methods). These are related in part (i) to the duration of a typical experiment (more than an hour on the same molecule), (ii) to the many motor rotation sequences (each associated with mechanical vibrations), and (iii) to the limited stability of the anchoring (full breakage or partial breakage, which eventually induces changes in the state of rotation, as in the situation of “rotation skid” (see Methods)). Nevertheless, the observation of localized blockages in Buff-B that are strictly associated with the presence of a mismatch was extremely reproducible, *e.g.*, approximately one hundred measurements were performed to gather the experimental data that are described in the next section. However, when data on many plectoneme-curve-segments of the same molecule was gathered, a rotation skid was often encountered at the surface anchoring of the molecule. When a rotation skid occurred, the zero reference rotation of the molecule was no longer obtained with the rotation value that was previously associated with R = 0; as a result, the rotation positions in which the plectonemes-formation-curves occur were shifted. Thus, the zero reference curve in Buff-B was also obtained, whenever possible, at the end of the experiment. In general, the repetition of experiments demonstrated that the determination of the position of the mismatches with respect to their neighbors was quite robust (within a turn). In addition, the following experimental characteristic signature pattern, which was found to be very robust, was found as a function of rotation for the molecular sequence that was tested in this study: an easily identified “gap” of fixed span (region R = −488 to −507 in Fig. 5) with no mismatch surrounded by very closely spaced mismatches on either side. This signature pattern was systematically observed for tens of molecules in the construct. However, we found that the absolute value of the mismatch position determination was often subject to much larger random errors (in the order of 1–20 turns) when no final reference curve could be gathered at the end of the experiment, which we associate with possible rotation skids of imperfect anchorages (see Methods).

### Kinetic Characteristics of Blockages

**Figure 6:**
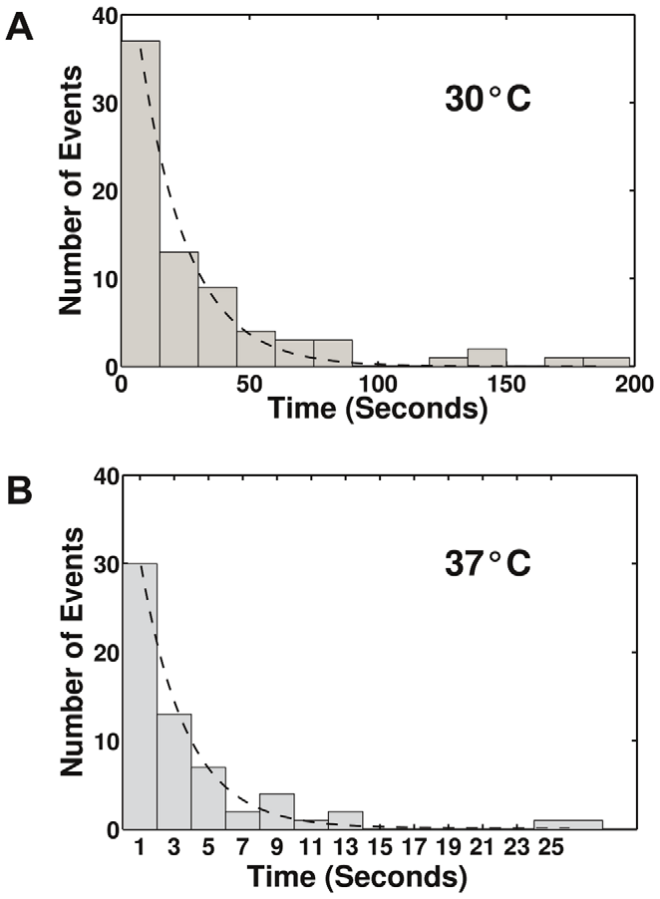
Waiting time for bypassing a blockage under “high stress” conditions. The construct used contains a single base difference between the test sequences (see Methods). The experiments were performed with a HJ that was formed in Buff-A and manipulated in Buff-B. The initial force was approximately 0.2 to 0.3 pN, and the rotation was adjusted to induce the formation of plectonemes after HJ blockage (20 negative turns after the blockage). The force was then quickly raised to approximately 4 pN (“high stress” conditions), although the rotation was unchanged. A mismatch bypass was observed after a variable time. The number of events observed for a given waiting time interval was plotted as a function of the waiting time. The data shown in Fig. 6A and Fig. 6B were obtained at temperatures of 30°C and 37°C, respectively. The data were fit assuming an exponential decay (continuous line); the characteristic times obtained were approximately 19 and 3 seconds at 30°C and 37°C, respectively.

When the typical operating protocol that was described earlier was used to explore the mismatch-induced blockages and the position of the magnets was left unchanged, the waiting time for an eventual crossing of the blockage by thermal fluctuation was excessive with respect to the molecule tethering breakage time. Let us consider a mismatch bypass with a low force (0.2 to 0.3 pN). When 20 supplementary negative turns were applied beyond the blockage (notation ΔR = −20) in an attempt to induce mismatch bypass, the waiting time for the bypass under these “low stress conditions” was typically in the range of one hour. However, we observed that this waiting time may be considerably reduced when “high stress” conditions (∼ 4 pN and ΔR = −20) were applied; thus, an efficient data gathering on the waiting times for mismatch bypass under “high stress” conditions may be performed (see Methods). We collected the corresponding waiting-time data on the bypass of a single sequence difference (see Methods for the construct used), and the data presented in Fig. 6 correspond to a series of experiments that were performed at two different temperatures (30°C and 37°C). A decreased waiting time was apparent when the temperature was increased. Exponential fits were performed on the distribution of the waiting times to evaluate the characteristic mismatch-bypass waiting times τ under high stress, which were found to be approximately 19 sec at 30°C and approximately 3 sec at 37°C. Assuming that the kinetics of the mismatch-bypass process of the HJ under high mechanical stress follow the Arrhenius law, the activation enthalpy was estimated to be approximately 80 kbT, where kb is the Boltzmann constant and T = 300 K (the absolute temperature), which corresponds to approximately 200 kJ/mol. We are not aware of any published experimental data with which we can compare this value (see Discussion). Under the “high stress” conditions used (approximately 4 pN and ΔR = −20 turns), the arms of the molecule under mechanical tension were nearly straight and the bead-to-surface distance approached the corresponding contour length L1. When the HJ was positioned at the mismatch, this length L1 was approximately 2.5 µm (see Methods). If one assumes a linear twist elasticity in the estimation of the torque Γ1 that is exerted on the molecule under “high stress” conditions, the torque can be expressed as Γ1 = kbT.2π.ΔR.C/L1, where C is the twist persistence length, which is on the order of 90 to 100 nm [21], [22], [23]; according to this equation, the torque is estimated to be Γ1 ∼ 20 pN nm. However, this value is not valid because it is beyond the critical torque Γ0 ∼ 10 pN nm that has been reported to induce the onset of a structural transition in DNA [25] upon negative winding. This transition is attributed to the formation of denatured DNA and/or some combination of non-canonical structures, such as Z-DNA [25]. Therefore, under “high stress” conditions, the molecule is expected to be in a regime in which the non-denatured DNA might coexist with the partly denatured DNA. Thus, under these experimental conditions, it is possible that a structure of the activated state for mismatch bypass might actually involve a partially denatured or altered junction. In contrast, the “low stress” conditions used in Figs. 4 and 5 for the determination of the blockage positions were performed under the limited torque Γplecto that is associated with plectoneme formation (as an estimate, Γplecto ∼ 3 pN nm at F = 0.2 pN and 500 mM NaCl [21]). Therefore, the nature of the activated state for mismatch bypass at low stress conditions and its characteristic activation energy might actually be very different from those at high stress.

## Discussion

A key experimental factor is the manipulation of the buffer composition, in which the concentration of magnesium ions influence the conformation and kinetics of the junction [12], [26], [27], [28], [29], [30]. In the absence of metal ions, the junction adopts an extended geometry in which the arms are directed toward the corners of a square with an open central region; moreover, the base-pairing is not maintained in the core of the correspondingly loose structure. When divalent ions are present at physiological concentrations, a stacked X junction is formed. This X junction structure involves a pair-wise coaxial stacking of the double-stranded helices that emerge from the HJ to form a right-handed, anti-parallel cross. The base-pairing and base-stacking are thought to be maintained even at the strand crossings. In the case of a non-mobile junction, there are two possible strand-stacking conformers that differ in the pair of helices that are stacked against each other, and a single junction can adopt both stacking conformers and exhibit a continued exchange between these [30]. In a mobile junction, a transient arm unstacking also occurs and it is believed that the activated state for junction migration (the exchange of two base pairs) occurs via a path that shares common characteristics with arm unstacking (observed in non-mobile junctions) [28]. The composition of the buffer is known to significantly influence the exchange between the stacking conformers and the HJ migration kinetics [27]. The activation enthalpy for junction migration reported by McKinney et al. (in 10 mM Tris-HCl, 50 mM MgCl2 and 50 mM NaCl at 25°C) is in the range of 10 to 40 kJ/mol [28]. One expects that this activation is substantially reduced when 1 mM MgCl2 is used instead of 50 mM MgCl2 (in the present experiment, the buffer contained 50 mM HEPES-KOH pH 8, 50 mM KCl, and 1 mM MgCl2 at 37°C), which results in easy migration. In fact, within our experimental resolution, the junction migration within a homologous region appeared to proceed smoothly when the rotation was changed, and the typical time to reach apparent equilibrium after a turn was found to be on the order of a fraction of a second. In addition to the simple effect that the buffer composition has on the migration kinetics, the force applied might also have additional influence. In the present experimental configuration, a force was applied on opposite arms of the junction, which tends to reduce the inter-helical angle Φ between the stacked pairs of helices of the HJ away from its expected value at zero force (Φ = 40° for a stacked-X junction at zero applied force). As a result, the application of a force might favor the path toward Φ = 0° (knowing that Φ = 0° is the value estimated for the transition state between the stacking conformers [30]). A stretching force (0.2 to 0.3 pN even in the low range) might facilitate the conformer exchange and possibly favor the junction migration kinetics.

We will now address the feature of migration blockage by the presence of a mismatch. As a surprising feature of the mismatch detection that was presented in this study, the blockages obtained for various single base-pair differences were found to be very robust and lasted a sufficiently long time such that we were able to obtain detailed data on the plectoneme formation curves. This effect enabled a good signal-to-noise determination of the presence and localization of the mismatches. We are unaware of any published data concerning the activation enthalpy associated with mismatch bypass in a HJ. In particular, the work of McKinney et al. [28] has not been extended to this type of situation. The measurements that we obtained for mismatch bypass under “high stress” conditions are likely not relevant to the mismatch bypass at “low stress” conditions, which were used for mismatch detection and mapping. Moreover, an intriguing and unexpected feature was uncovered in the present work: the ratchet-like behavior of the Holliday junction, which exhibits easy mismatch resorption and prevents mismatch formation at the junction when the proper ionic conditions are present. We find this ratchet behavior counterintuitive because, even if it is evident that the final state (mismatches disappeared) is more stable, it is unclear why the transient activated state associated with mismatch resorption has a lower free energy than the free energy associated with the transient activated state for mismatch formation. This unexpected finding might be caused by the mismatch formation following a different path than that followed during mismatch resorption (*e.g.*, an effect that possibly results from changes in the strand stacking conformation and kinetics, which are induced by the presence of mismatches close to the core of the junction). This ratchet-like behavior actually enables the sequential data gathering that was demonstrated in this study.

The present work demonstrates that a mapping of the differences between two DNA strands can be obtained without the need for any a priori knowledge of the sequences. In the case studied, a map of a handful of the differences in the region to be tested was established. If two mismatches were close together, the differentiation between the two mismatches became difficult and eventually impossible. Another drawback of the method presented is the necessity to combine the two sequences that will be compared in a single molecule. The magnetic tweezers assay may be parallelized, as already demonstrated [31] with approximately 400 molecules. We also speculate that the use of a HJ as a discriminating device to compare the two sequences might also be adapted to a nanopore method if a strand of the HJ, but not the junction itself, is able to be translocated trough the pore. If that is the case, the translocation would be coupled to the HJ migration with the expectation that the transient HJ blockages that act as a molecular brake could induce detectable transient modifications in the current.

### Conclusion

In conclusion, the present experiment demonstrates that HJ migration enables the detection and mapping of single base pair differences between two several-kb-long sequences. The behavior of a Holliday junction with mismatches is intriguingly akin to the “sand in a gearbox” paradigm, in which the Holliday junction plays the role of the gearbox and the differences in the sequences assume the role of the grains of sand; as an unexpected added twist, the mechanical properties that are associated with this DNA gearbox are sensitive to the direction of rotation.

## Methods

The buffers used were Buff-A (25 mM Tris-acetate pH 8, 0.5 mM EDTA, 1 mM DTT, 1 mg/ml BSA, and 1 mM NaN3) and Buff-B (50 mM HEPESKOH pH 8, 50 mM KCl, 1 mM MgCl2, 1 mM DTT, 1 mg/ml BSA, and 1 mM NaN3), and the sample temperature was 37°C.

### Molecular Construct

The construct was originated from a plasmid that was described previously [15] with a fragment of lambda-phage DNA; this DNA comprises a region from a HindIII site to a KpnI site that was cloned into a HindIII/KpnI-digested pksII plasmid. Then, the naturally occurring oxa gene (7 or 11) was cloned into the BlpI site of the resulting plasmid. Each arm of the resulting construct comprises the 6.6 kb fragment that was formed from the BsaI-HindIII digestion of the previously described plasmid. Multiple digoxigenin and biotin labels (for the arm containing the oxa gene 7 and 11, respectively) were introduced [15] on one extremity (proximal to the BsaI cut). The DNA construct (with a total length of 13.2 kb) was then obtained via the head-to-head ligation (using the HindIII cut overhang) of the two similar 6.6-kb DNA fragments that were described, each of which contains a different oxa gene. The DNA sequence on each side of the center of the construct was quasi-palindromic. The construct was anchored between a paramagnetic Dynal M280 streptavidin-coated bead (with a 2.8-µm diameter) and the internal surface of a rectangular capillary tube (used to contain the sample) that had been coated with antibodies against digoxigenin [15].

### Estimation of the Force

Using the equipartition theorem, the lateral Brownian fluctuations of the bead may be analyzed to deduce the vertical force exerted on the molecule [14]. Once the vertical position of the magnets is chosen, the vertical force applied is nearly constant because variations in the length of the construct (a few micrometers) are negligible with respect to the magnet-to-sample distance (of at least one millimeter). A given vertical position of the magnets does not warrant a given traction force because of the bead-to-bead variations in the magnetic properties. Nevertheless, if a force-calibration has not been achieved successfully for a particular bead/molecule at the chosen vertical magnet position (*e.g.*, because of premature breakage of the tethering), the vertical force may be estimated a posteriori as follows. The effective number of turns of the molecule is estimated (see Detailed Numerical Methods) such that (i) a correspondence can be established between the rotation R and the relative winding σ and (ii) the effective curvilinear length L0 of the molecule may be estimated. The maximum height H of the plectoneme formation curve (at R = 0) divided by L0 gives the (force-dependent) intrinsic relative height. This value is then compared to the values for the plectoneme reference curves that were obtained at a series of forces in the same buffer but for a different molecule; an estimation of the force may thus be obtained. For confirmation, the width at the mid-height of the plectoneme formation curve (relative height as a function of the relative winding σ) is also force-dependent and may be compared in a similar fashion (*e.g.*, see the features present in [21]).

### Exploring a Range of Sequences and Testing for the Presence of Mismatches

The motor that rotates the magnets above the sample and thus rotates the beads has a speed of 6 turns/sec. Two types of measurement cycles were typically used: “fast” and “slow”. The speed of the motor was the same in both modes, and the difference between the modes is the waiting time between the rotation increments during which the bead-height data are acquired (see later). The total cycle time, including the motor rotation and the waiting time, is 2.5 and 10 seconds in the fast mode and the slow modes, respectively. The fast mode was used for fast but noisier measurements, whereas the slow mode was used for slower but less noisy measurements. The details of each bead-height measurement are the following: for a single change in rotation, the motor is first activated toward the next rotation value, a bead height measurement it then performed at the video rate and the average measurement is stored in a file for subsequent analysis. In the slow mode, after the motor motion has been started, there is a waiting time of approximately 2 seconds; the measurement then proceeds for approximately 8 seconds. To explore an unknown region starting from a given R = R1 (in the migrating HJ regime), the fast mode was first used. The value of R was then decreased sequentially by 1-turn steps for approximately 12 to 15 turns, and a characteristic decrease in the height was monitored to determine whether the curve was distinct from the previous one, thus indicating a different migration blockage. If such distinct decrease was observed, R was returned to its initial value R1 and the slow mode was used for detailed data acquisition, which involves the decreasing of R in one-turn steps for approximately 20 to 30 steps. Once the region was explored, R was increased continuously to 2 turns more than the last maximum value R1 that was explored, and a test for the presence of a new plectoneme-curve-segment was performed by again decreasing R in a stepwise manner in the fast mode. The same process was repeated until all detectable plectoneme-curve-segments in the region of interest were gathered. This method, which alternates between the fast and slow modes, has the advantage of decreasing the total time of data gathering and the disadvantage of relying only on the noisier fast mode for the detection of novel plectoneme-curve-segments.

### Overview of the Numerical Methods

Two parameters were determined: the change in the rotation (offset Ri) and the corresponding estimated number of turns Ni between the end-anchoring in the torsion-relaxed molecule that is shortened because of the HJ progression and the blockage. Using MatLab, a smooth interpolation curve Cref-fitted was first determined using the experimental Cplecto data (acquired in Buff-B at the end of the experiment) and segmented cubic splines (see Detailed Numerical Methods). This interpolation expresses the relative height with respect to the height at R = 0 as a function of the relative rotation σ. Then, for each curve-segment Bplecto of the plectoneme formations obtained, a non-linear minimization algorithm (Levenberg-Marquardt) was used to determine the best Ri and Ni that adjusts the Cref-fitted (see Detailed Numerical Methods). The values Ri were then compared with the theoretical values for the encounter of the HJ with mismatches obtained from the sequence as follows: from the center of symmetry of the construction, the base indices where the sequence differences occur are multiplied by 2 and divided by 10.45, which is the estimated helical repeat of DNA [24]. The factor of 2 is used because 2 turns of the bead are necessary to progress by one turn in each arm. To estimate the errors in the parameter determination, a bootstrap method was used (see Detailed Numerical Methods).

### Detailed Numerical Methods

The plectoneme reference curve of the bead-height as a function of the rotation R was obtained in Buff-B at the end of the experiment, with small rotation values. This reference curve is a bell-shaped curve. To obtain a normalized Cref curve that expresses the normalized height (normalized to one at the zero rotation) as a function of the relative winding σ of the molecule, the following procedure was used: the experimental height was first divided by the maximum height of the curve H0 (the rotation at the maximum height defines R = 0), and the rotation values were divided by the total number of turns N0 in the torsion-relaxed construction, *i.e.*, the effective number of turns between the anchoring points of the construct. The value of N0 is related to the number of bases between the anchoring points of the construct divided by the helical repeat of DNA, which is taken to be 10.45 [24]. This number of bases may be nearly equal to or smaller than the total number of bases in the construct because the end-labeling of the anchoring may span an unknown length (typically smaller than 1 kb). In practice, the value of N0 is determined using a linear fit of the curve of the height as a function of the rotation that is obtained in a migrating HJ regime. The intercept (when the height equals zero) gives an estimate of N0. The data shown in Fig. 5 were obtained with a molecule in which N0 is approximately 1,200 (to be compared to the maximum expected value of 1,250) and the maximum height H0 was determined to be approximately 2.76 under the conditions of the experiment. These values were used to normalize the plectoneme reference curves. To fit the normalized reference curve, a segmented cubic spline fit Cref-fitted (see Fig. S1-A) was constructed using MatLab and the function splinefit and imposing a mirror symmetry on the fit (source code for splinefit: www.mathworks.com/matlabcentral/fileexchange/13812-splinefit). A function was then determined using Ri (putative blocking rotation) and Ni (putative number of turns between the tethering in the torsion-relaxed shortened blocked molecule) as the arguments. This function (a) divides the experimental heights by H0*Ni/N0 to obtain the relative height data, (b) subtracts Ri from the rotation data and divides the resulting value by Ni to obtain the relative rotation data and (c) expresses the residuals between the experimental data and the reference data Cref-fitted. A Levenberg-Marquardt algorithm was then used to determine the best parameters for minimizing the sum of the squared residuals. The Levenberg-Marquardt function used is based on lmfnlsq (source code for lmfnlsq: www.mathworks.com/matlabcentral/fileexchange/17534-lmfnlsq-solution-of-nonlinear-least-squares using MatLab). We found that the initial fitting procedure that is described above encountered a problem: the shapes of the plectonemes-curve-segments differed somewhat from the reference curves at lower heights. We assumed that this problem was an effect of the size of the bead. Thus, the following simple procedure was used to circumvent this problem: a weight factor (exp{3 * [(relative height) −1]}) was used to multiply the residuals between the data and the fit such that the fitting was stringent for the top part of the curves and less stringent for the deviations in the lower parts of the curve (see Fig. S1-B for an example of the fit of a plectoneme-curve-segment). To estimate the errors in the fitted parameters, a bootstrap method was used. However, because a weight factor was used in the fitting procedure, the following simple scheme was chosen: all sections in the upper half of the blocking plectonemes-curve-segments were used to collect the (un-weighted) residuals between the fits and the data and as the “raw residuals” in the bootstrap method. For each plectoneme-curve-segment ui and its corresponding fit ûi, the simulated data u’i were obtained by adding randomly chosen residuals from the raw residuals file to ûi. Then, the fitting procedure was performed to obtain û’i and an estimated blocking rotation R’i. This procedure was repeated 1000 times for each plectoneme-curve-segment. The standard deviation SD of R’i was found to be in the range of 0.5 to 0.6 turns, depending on the plectoneme-curve-segment, whereas the average values of R’i were slightly increased (approximately 0.3 to 0.4 turns) with respect to the initial fitted values (before bootstrapping). We have also explored how a change in the parameter N0 affects the Ri values. In fact, N0 is involved (in the expression of σ) both in the reference curve and in the fitted plectoneme-curve-segments; thus, the effects are canceled out. The changes in the fitted Ri for the data shown in Fig. 5 were smaller than 10^−2^ turns when N0 was arbitrarily changed from 1,200 to 1,000.

### Limitation in the Resolution of a Mismatch when Mismatches are Close Together

When comparing the blockages detected and the list of mismatches in the region covered (see Sequence Information S1), it appeared that the described procedure failed to detect all individual mismatches when the mismatches were very close together. One reason for this failure stems from the following. Let us consider two mismatches m and n with expected rotation blockages Rm and Rn (Rm<Rn). If the experimental run does not include a backward exploration that starts between Rm and Rn, then it is not possible to differentiate between m and n. In addition, because the experimental run time was limited by the anchoring stability of the molecule, only coarse-grained initiations of backward exploration series were performed. Furthermore, the detection of novel plectoneme-curve-segments was only based on the noisier “fast” mode.

### Activation Energy Associated with Blockage Bypass under “High Stress” Conditions

Under “high stress” conditions, a different molecular construct with a single sequence difference (one additional AT base pair in one sequence compared to the other, which results upon recombination in a T-bulge and an A-bulge) was used. The total length of the construct was 13.2 kb (identical length to the construct used for Figs. 3 through 5), and the base-index for the locus with the difference was 2386 (counted from the center of symmetry of the molecule). When the HJ was positioned near this mismatch, the corresponding number of base pairs involved in the length associated with the bead tethering, i.e., excluding the side-arms of the junction, was approximately 8.4 kb (13.2 kb - (2 * 2.4 kb)), which corresponds to a curvilinear length of approximately 2.5 µm. We evaluated the activation barrier that opposed the HJ progression in the presence of this mismatch in Buff-B. In Buff-B, the HJ under low traction force (0.2 to 0.3 pN) was positioned just before the mismatch formation; then, 20 negative turns were applied beyond this HJ blockage (noted ΔR = −20). To obtain faster and experimentally tractable kinetics, the force applied to the construction was then quickly increased from an initial low traction force (0.2 to 0.3 pN, ΔR = −20) to approximately 4 pN (magnet rotation was unchanged). After a variable time, a small and abrupt jump was observed in the data of the bead-height as a function of time. During preliminary experiments, it was verified that resetting the force to the initial low value after this jump resulted in further unrestricted HJ motion for negative migration. This finding implies that the jump is effectively associated with the bypass of the mismatch blockage.

### Experimental Difficulties

A difficulty that was often encountered was the limited lifetime of the molecular construct and its anchoring: (i) upon breakage of the construct, which most likely occurred at one of its anchoring extremities, the experiment was terminated (this problem became more acute because the time required to perform all of the measurements is long, i.e., the measurements were obtained in the course of more than one hour if many plectoneme-curve-segments were to be collected); (ii) in some instances, the length of the molecule decreased irreversibly after a cycle of R manipulation, which possibly corresponds to a partial adsorption of one or both tethering arm(s) on the surface(s); and (iii) sometimes during an experimental run, the zero rotation (deduced from the maximum value of the bead-height with no HJ present) was found to have changed between the beginning and the end of the experimental run. During the experiment, under the rotational stress and the repeated sequences of motor rotation, each of which is associated with mechanical vibration, a change in the reference zero rotation is likely due to a transient loss of the anchoring on one strand at one extremity of the construct, followed by partial rotational stress relief and subsequent re-establishment of the anchoring, which results in a “rotation skid”. To identify whether these problems occurred, the plectoneme reference curves were systematically acquired at the beginning of the experiments and, whenever possible, re-acquired at the end of the experiments.

## Supporting Information

Figure S1 **Example of fits.** Fig. S1-A: Segmented spline fit of the experimental data corresponding to a plectonemes formation curve. The relative height was plotted as a function of the relative winding σ. Fig. S1-B: Illustration of the fit of the data obtained with an experimental blockage. The fit was optimized for the upper section of the curve (see Detailed Numerical Methods) and is tolerant to errors in the lower part.
(PDF)

Sequence Information S1. (PDF)
