# Supplementary material for: Mapping of Single-Base Differences between Two DNA Strands in a Single Molecule Using Holliday Junction Nanomechanics
Source: PLoS One. 2013 Feb 5;8(2):e55154. doi: 10.1371/journal.pone.0055154 (PMC3564857; doi:10.1371/journal.pone.0055154)
Supplement: Figure S1 — Example of fits. Fig. S1-A: Segmented spline fit of the experimental data corresponding to a plectonemes formation curve. The relative height was plotted as a function of the relative winding σ. Fig. S1-B: Illustration of the fit of the data obtained with an experimental blockage. The fit was optimized for the upper section of the curve (see Detailed Numerical Methods) and is tolerant to errors in the lower part. (PDF) [file pone.0055154.s001.pdf]

SuppFig1

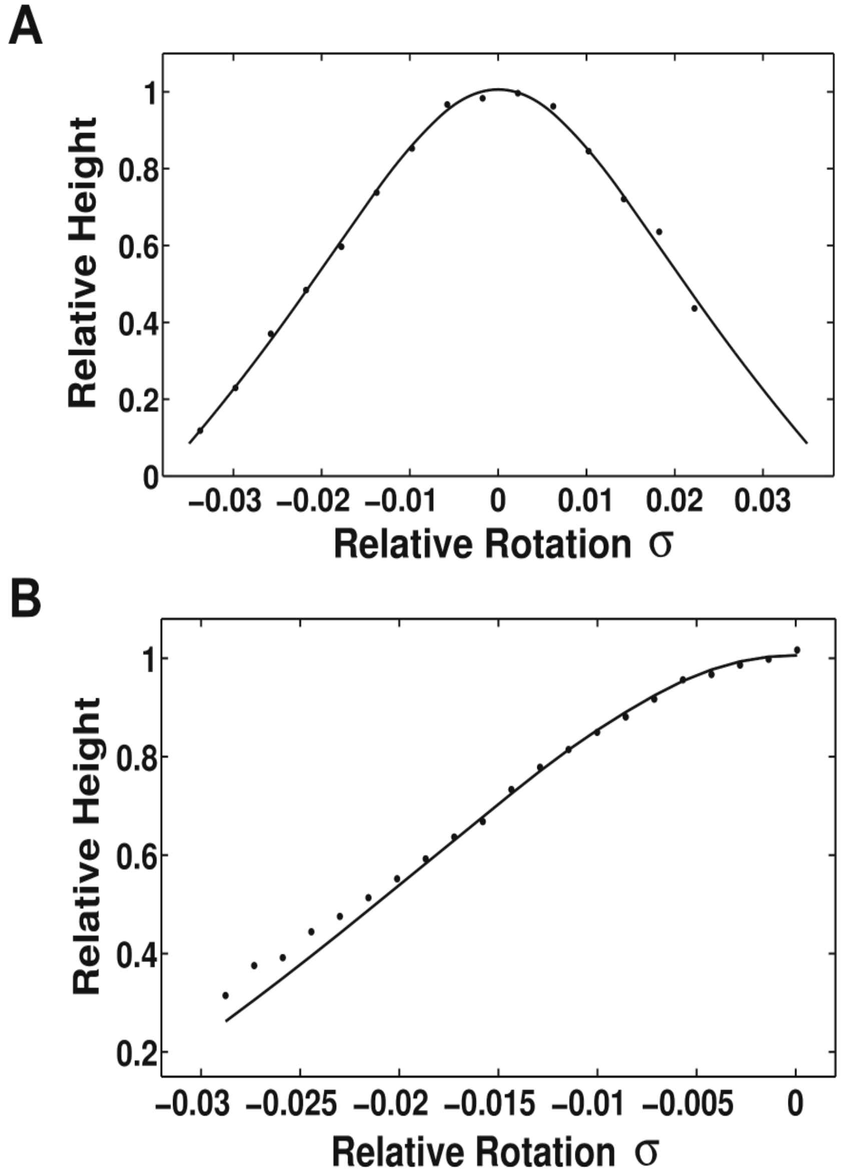

Legend of supplementary figure S1-A and S1-B:

Example of fits: the figure S1-A shows a segmented spline fit of the experimental data corresponding to the plectonemes formation curve, where relative height is plotted as a function of relative winding σ. The figure S1-B is an illustration of a fit performed on an experimental blockage. The fit has been optimized for the upper part of the curve (see Detailed numerical methods) and is tolerant to errors in the lower part.
