# Supplementary material for: Mapping of Single-Base Differences between Two DNA Strands in a Single Molecule Using Holliday Junction Nanomechanics
Source: PLoS One. 2013 Feb 5;8(2):e55154. doi: 10.1371/journal.pone.0055154 (PMC3564857; doi:10.1371/journal.pone.0055154)
Supplement: Sequence Information S1. — (PDF) [file pone.0055154.s002.pdf]

<u>Supplementary Sequence information</u>:

Sequence alignment of the regions where the two arms of the construction differ (Oxa7 vs. oxa 11). The indices are relative to the center of the construction. The numbers in bold are the indices corresponding to fig 5A and 5B

```
Oxa7 arm        GTTCGCAATCTGGGTATTGTTTGTTCCCCTGAGCGCGGTTGGTGCTGTTGGCGTTCCGGT 2280
Oxa11 arm       GTTCGCAATCTGGGTATTGTTTGTTCCCCTGAGCGCGGTTGGTGCTGTTGGCGTTCCGGT 2280
                ************************************************************

Oxa7 arm        CAGTGCCGGACTGTCCAGTGGGCTTTTCTGTTCGTTTCATCCATTACCACCTTAACCGCC 2340
Oxa11 arm       CAGTGCCGGACTGTCCAGTGGGCTTTTCTGTTCGTTTCATCCATTACCACCTTAACCGCC 2340
                ************************************************************
                                                            2386
Oxa7 arm        TTTGGCGTTGCAGCAAGCGTTTCAGACGTGCTGTTGGTTGCACTGCT--GAAAACATTTG 2398
Oxa11 arm       TTTGGCGTTGCAGCAAGCGTTTCAGACGTGCTGTTGGTTGCACTGCTATGAAAACATTTG 2400
                ***********************************************  ***********

                          2414             2427            2443
Oxa7 arm        CCGCATATGTAATTACTGCGTGTCTTTCAAGTACGGCATTAGCTAGTTCAATTACAGAAA 2458
Oxa11 arm       CCGCATATGTAATTATCGCGTGTCTTTCGAGTACGGCATTAGCTGGTTCAATTACAGAAA 2460
                ***************  *********** *************** ***************

                    2465                                     2505
Oxa7 arm        ATACGTTTTGGAACAAAGAGTTCTCTGCCGAAGCCGTCAATGGTGTTTTCGTGCTTTGTA 2518
Oxa11 arm       ATACGTCTTGGAACAAAGAGTTCTCTGCCGAAGCCGTCAATGGTGTCTTCGTGCTTTGTA 2520
                ****** *************************************** *************

                                           2548
Oxa7 arm        AAAGTAGCAGTAAATCCTGCGCTACCAATAACTTAGCTCGTGCATCAAAGGAATATCTTC 2578
Oxa11 arm       AAAGTAGCAGTAAATCCTGCGCTACCAATGACTTAGCTCGTGCATCAAAGGAATATCTTC 2580
                ***************************** ******************************

Oxa7 arm        CAGCATCAACATTTAAGATCCCCAACGCAATTATCGGCCTAGAAACTGGTGTCATAAAGA 2638
Oxa11 arm       CAGCATCAACATTTAAGATCCCCAACGCAATTATCGGCCTAGAAACTGGTGTCATAAAGA 2640
                ************************************************************

                         2650                                2685
Oxa7 arm        ATGAGCATCAGATTTTCAAATGGGACGGAAAGCCAAGAGCCATGAAACAATGGGAAAGAG 2698
Oxa11 arm       ATGAGCATCAGGTTTTCAAATGGGACGGAAAGCCAAGAGCCATGAAGCAATGGGAAAGAG 2700
                *********** ********************************** *************

                    2705                        2733
Oxa7 arm        ACTTGAGCTTAAGAGGGGCAATACAAGTTTCAGCGGTTCCCGTATTTCAACAAATCGCCA 2758
Oxa11 arm       ACTTGACCTTAAGAGGGGCAATACAAGTTTCAGCTGTTCCCGTATTTCAACAAATCGCCA 2760
                ****** *************************** *************************
```

```
                                           2790           2805    2811 2813
Oxa7 arm        GAGAAGTTGGCGAAGTAAGAATGCAGAAATATCTTAAAAAATTTTCATATGGTAACCAGA 2818
Oxa11 arm       GAGAAGTTGGCGAAGTAAGAATGCAGAAATACCTTAAAAAATTTTCCTATGGCAGCCAGA 2820
                ******************************* ************** ***** * *****

                                              2853 2855
Oxa7 arm        ATATCAGTGGTGGCATTGACAAATTCTGGTTGGAGGGTCAGCTTAGAATTTCCGCAGTTA 2878
Oxa11 arm       ATATCAGTGGTGGCATTGACAAATTCTGGTTGGAAGACCAGCTTAGAATTTCCGCAGTTA 2880
                ********************************** *  **********************

                                        2906                  2928   2937
Oxa7 arm        ATCAAGTGGAGTTTCTAGAGTCTCTATTTTTAAATAAATTGTCAGCATCAAAAGAAAATC 2938
Oxa11 arm       ATCAAGTGGAGTTTCTAGAGTCTCTATATTTAAATAAATTGTCAGCATCTAAAGAAAACC 2940
                *************************** ********************* ******** *

                                       2973  2976       2988
Oxa7 arm        AGCTAATAGTAAAAGAGGCTTTGGTAACGGAGGCTGCGCCTGAATATCTTGTGCATTCAA 2998
Oxa11 arm       AGCTAATAGTAAAAGAGGCTTTGGTAACGGAGGCGGCACCTGAATATCTAGTGCATTCAA 3000
                ********************************** ** *********** **********

                                                                       3057
Oxa7 arm        AAACTGGTTTTTCTGGTGTGGGAACTGAGTCAAATCCTGGTGTCGCATGGTGGGTTGGTT 3058
Oxa11 arm       AAACTGGTTTTTCTGGTGTGGGAACTGAGTCAAATCCTGGTGTCGCATGGTGGGTTGGGT 3060
                ********************************************************** *

                            3071
Oxa7 arm        GGGTTGAGAAGGGAGCAGAGGTTTACTTTTTCGCCTTTAACATGGATATAGACAACGAAA 3118
Oxa11 arm       GGGTTGAGAAGGAGACAGAGGTTTACTTTTTCGCCTTTAACATGGATATAGACAACGAAA 3120
                ************   *********************************************

                3119                                    3161
Oxa7 arm        ATAAGTTGCCGCTAAGAAAATCCATTCCCACCAAAATCATGGCAAGTGAGGGCATCATTG 3178
Oxa11 arm       GTAAGTTGCCGCTAAGAAAATCCATTCCCACCAAAATCATGGAAAGTGAGGGCATCATTG 3180
                 ***************************************** *****************

                      3187
Oxa7 arm        GTGGCTAAGAGCTGCACTATCCCCTTTCTCGTTGTGTCCGCATCCTCAAGCGCGACAGCT 3238
Oxa11 arm       GTGGCTAA-AGCTGCACTATCCCCTTTCTCGTTGTGTCCGCATCCTCAAGCGCGACAGCT 3239
                ******** ***************************************************
```
